# Blood Lipid Polygenic Risk Score Development and Application for Atherosclerosis Ultrasound Parameters

**DOI:** 10.3390/biomedicines12122798

**Published:** 2024-12-10

**Authors:** Marija Zaicenoka, Alexandra I. Ershova, Anna V. Kiseleva, Anastasia V. Blokhina, Vladimir A. Kutsenko, Evgeniia A. Sotnikova, Anastasia A. Zharikova, Yuri V. Vyatkin, Maria S. Pokrovskaya, Svetlana A. Shalnova, Vasily E. Ramensky, Alexey N. Meshkov, Oxana M. Drapkina

**Affiliations:** 1 National Medical Research Center for Therapy and Preventive Medicine, Ministry of Healthcare of the Russian Federation, 10-3, Petroverigsky per., 101000 Moscow, Russia; alersh@mail.ru (A.I.E.); sanyutabe@gmail.com (A.V.K.); blokhina0310@gmail.com (A.V.B.); sotnikova.evgeniya@gmail.com (E.A.S.); azharikova89@gmail.com (A.A.Z.); vyatkin@gmail.com (Y.V.V.); mpokrovskaia@list.ru (M.S.P.); sshalnova@gnicpm.ru (S.A.S.); ramensky@gmail.com (V.E.R.); meshkov@lipidclinic.ru (A.N.M.); drapkina@bk.ru (O.M.D.); 2 Moscow Center for Advanced Studies, 20 Kulakova Str., 123592 Moscow, Russia; 3 Faculty of Bioengineering and Bioinformatics, Lomonosov Moscow State University, 1-73, Leninskie Gory, 119991 Moscow, Russia; 4 Institute for Artificial Intelligence, Lomonosov Moscow State University, 1-73, Leninskie Gory, 119991 Moscow, Russia; 5 National Medical Research Center for Cardiology, 15A, 3-ya Cherepkovskaya Str., 121552 Moscow, Russia; 6 Research Centre for Medical Genetics, 1 Moskvorechye Str., 115522 Moscow, Russia; 7 Department of General and Medical Genetics, Pirogov Russian National Research Medical University, 1 Ostrovityanova Str., 117997 Moscow, Russia

**Keywords:** low-density lipoprotein cholesterol, high-density lipoprotein cholesterol, total cholesterol, triglycerides, polygenic risk scores, atherosclerosis, cardiovascular disease

## Abstract

**Background:** The present study investigates the feasibility of using three previously published genome-wide association studies (GWAS) results on blood lipids to develop polygenic risk scores (PRS) for population samples from the European part of the Russian Federation. **Methods:** Two population samples were used in the study – one from the Ivanovo region (*n* = 1673) and one from the Vologda region (*n* = 817). We investigated three distinct approaches to PRS development: using the straightforward PRS approach with original effect sizes and fine-tuning with PRSice-2 and LDpred2. **Results:** In total, we constructed 56 PRS scales related to four lipid phenotypes: low-density lipoprotein cholesterol, high-density lipoprotein cholesterol, total cholesterol, and triglyceride levels. Compared with previous results for the Russian population, we achieved an additional R^2^ increase of 2–4%, depending on the approach and lipid phenotype studied. Overall, the R^2^ PRS estimates approached those described for other populations. We also evaluated the clinical utility of blood lipid PRS for predicting carotid and femoral artery atherosclerosis. Specifically, we found that PRS for total cholesterol, low-density lipoprotein cholesterol, and triglycerides were positively correlated with ultrasound parameters of carotid and femoral artery atherosclerosis (ρ = 0.09–0.13, *p* < 0.001), whereas PRS for high-density lipoprotein cholesterol were inversely correlated with the number of plaques in the femoral arteries (ρ = −0.08, *p* = 8.71 × $10^{-3}$). **Conclusions:** PRS fine-tuning using PRSice-2 add LDpred2 improves the performance of blood lipid PRS. Our study demonstrates the potential for further use of blood lipid PRS for prediction of atherosclerosis risk.

## 1. Introduction

Polygenic risk scores (PRS) are based on genome-wide association study (GWAS) results [1] and allow one to estimate genetic liability of an individual to complex phenotype development. PRS can serve as prognostic markers for personalized disease prevention [2,3] by early identification of at-risk individuals with high polygenic risk, in some cases comparable to monogenic variants [4].

Several factors may contribute to the limited transferability of PRS between populations [5]. First, risk allele frequency, linkage, and effect size may differ across populations [6]. Second, trait heritability is influenced by both the above-mentioned genetic architecture of a population [7] and non-genetic factors such as age, sex, and socioeconomic status [8]. These variables can significantly impact the accuracy of risk prediction, even within the same ancestral group [9]. Besides relatively few recent studies [10,11,12,13], the Russian population remains underrepresented in large genome studies; thus, the question of PRS transferability to estimate individual risks in this population remains largely open [14].

Blood lipids are a significant and modifiable risk factor for atherosclerotic cardiovascular diseases [15], which are the leading cause of death worldwide [16]. Circulating blood lipid levels include, among others, high-density lipoprotein cholesterol (HDL-C), low-density lipoprotein cholesterol (LDL-C), total cholesterol (TC), and triglycerides (TG). The heritability of LDL-C, HDL-C, TC and TG has been shown to range from moderate (20%) to high (60%) [17], indicating that the genetic component plays a substantial role in explaining interindividual variations in lipid phenotypes [18,19]. PRS may be used not only for GWAS target phenotypes, but also for related phenotypes [1]. Cholesterol is a key component of atherosclerotic plaque. All the apoB-containing lipoproteins (both LDL-C and triglyceride-rich remnant lipoproteins) play a crucial role in all stages of atherosclerosis development, from the fatty streak phase to vulnerable plaque [20]. Infiltration and retention of apoB-containing lipoproteins in the artery wall is a critical initiating event that triggers an inflammatory response and atherosclerosis [21]. Epidemiological studies have clearly established an inverse relationship between HDL-C levels and risk of atherosclerotic cardiovascular diseases [22]. Numerous studies have evaluated blood lipid PRS [23], linking blood lipid PRS to coronary artery disease [24,25,26], familial hypercholesterolemia [27,28,29], and cardiovascular disease treatment effectiveness [30], suggesting their possible clinical utility. The ultrasound parameters of carotid and femoral atherosclerosis are valuable markers for estimating subclinical atherosclerosis and cardiovascular risk [31]. Despite the established genetic component of atherosclerosis [32], the number of studies evaluating the possible clinical utility of PRS (and blood lipid PRS, specifically) for subclinical atherosclerosis remains limited. The quantile of TC-related PRS value was demonstrated to be moderately associated with carotid intima-media thickness (IMT) in adult women (*p* = 0.0182), regardless of principal components and age [17]. Another study has shown that, after adjustment for age and sex, all four lipid PRS (TC, LDL-C, HDL-C, and TG) were associated with carotid plaques. This relationship was the strongest for the LDL-C score, which increased plaque score by 0.102 per standard deviation (SD) increase in PRS (*p* = $3.2\times {\mathrm{10}}^{-8}$) [33]. In a study investigating the contribution of genetic cardiometabolic risk factors to carotid plaque formation in type 2 diabetic patients and healthy controls, HDL-C PRS was inversely associated with carotid IMT (−0.01 SD per allele, *p* = 0.034) and plaque formation (−0.01 SD per allele, *p* = 0.036), whereas LDL-C PRS was positively associated with carotid IMT (0.01 SD per allele, *p* = 0.039) [34]. This area of study is still in its early stages and demands more comprehensive research to systematically evaluate the relationship between blood lipid polygenic risk scores (PRS) and atherosclerosis ultrasound parameters.

This study aims to evaluate the applicability of existing blood lipid GWAS findings for constructing PRS tailored to the Russian population. By utilizing various PRS development methods, we also aim to assess their clinical potential in predicting atherosclerotic phenotypes.

## 2. Materials and Methods

### 2.1. Selection of Participants

We used data collected during the “Epidemiology of Cardiovascular Diseases and Risk Factors in Regions of the Russian Federation” (ESSE-RF) cross-sectional study [35], namely the two representative samples for which the next-generation sequencing (NGS) data were available: ESSE-Ivanovo (*n* = 1858) [11,36] and ESSE-Vologda (*n* = 850) [37]. The two regions have close ethnic background (95.9% and 96.5% Russians in the Ivanovo and Vologda regions, respectively) and socioeconomic characteristics [38]. Quality control included the following steps: removal of relatives, PCA outlier removal by means of hierarchical clustering, and removal of samples with lacking blood lipid phenotype or covariate data.

### 2.2. Clinical Data

The data from ESSE-RF included sex, age, body mass index (BMI), smoking status (never smoked, ex-smokers, current smokers), and statin intake.

The lipid levels (LDL-C, HDL-C, TC, and TG) were determined using the Abbott Architect C-8000 system (Abbott Laboratories, North Chicago, IL, USA) and reported in mmol/L [39]. Triglyceride levels were logarithmized for further analysis.

The ultrasound parameters were included in the analysis and obtained as part of the ATHEROGEN-Ivanovo substudy conducted in the Ivanovo region between 2013 and 2015 as part of the larger ESSE-RF study [40]. As part of the ATEROGEN-Ivanovo, participants in the ESSE-Ivanovo cohort, aged 40–67 years, underwent ultrasound examination of the carotid and femoral arteries 2–4 years after enrollment in the ESSE-RF study. High-resolution B-mode ultrasonography was performed with a 12–5 MHz linear probe (MySono U6, Samsung Medison, Seoul, Republic of Korea). All measurements were performed in both common carotid arteries (CCA), available for ultrasound visualization proximal segments of the internal carotid arteries (ICA), both common femoral arteries (CFA), and 1.5 cm of the proximal segments of the superficial femoral arteries (SFA). All measurements were made in diastole, corresponding to the R-wave of the electrocardiogram [41]. We included the following ultrasound parameters from ATHEROGEN-Ivanovo: IMT on both sides, plaque number, maximum stenosis, total stenosis, and plaque score for carotid and femoral arteries. IMT measurements were made in accordance with a Consensus Statement from the American Society of Echocardiography Carotid Intima-Media Thickness Task Force (2008) [42].

The presence of plaques was estimated at six sites of the carotid arteries and six sites of the femoral arteries: the total length of both CCAs (CFAs), both carotid (femoral) bifurcations, and both proximal segments of ICAs (SFAs). A plaque was defined as a focal structure that encroaches into the arterial lumen of at least 0.5 mm, or 50% of the surrounding IMT value, or demonstrates a thickness ≥1.5 mm measured from the media-adventitia interface to the intima–lumen interface [41]. Plaque number was defined as the sum of all plaques.

Percent diameter stenosis was defined at the site of maximum plaque obstruction in the artery in the transverse view and was obtained from measurements of the residual lumen diameter and the original diameter [42]. The maximum stenosis is the maximum value of all percent diameter stenoses of carotid (femoral) arteries. Total stenosis is the sum of all maximum stenoses of carotid (femoral) arteries.

For plaque score measuring, each carotid (femoral) artery was divided into four segments: 15 mm of proximal ICAs (SFAs) after the tip of the flow divider, the carotid bulb, and two distal parts of the CCAs (CFAs), each 15 mm. The plaque score was calculated by summing the maximum thickness of all plaques measured in millimeters on the near and far walls at each of the four parts of both sides of the carotid (femoral) arteries [43].

### 2.3. Ethics Statement

The ESSE-RF study was conducted according to the ethical provisions of the Declaration of Helsinki and the National Standard of the Russian Federation “Good Clinical Practice (GCP)” GOST R52379-2005. The study was approved by the Independent Ethics Committee of the National Medical Research Center for Therapy and Preventive Medicine (Protocol number 07-03/12 from 3 July 2012). Each participant provided their written informed consent to take part in this scientific project.

### 2.4. DNA Extraction and Sequencing

Genomic DNA was extracted from peripheral blood samples with the use of the QIAamp DNA Blood Mini Kit (Qiagen, Hilden, Germany). The DNA concentration was determined using a Qubit 4 fluorometer (Thermo Fisher Scientific, Waltham, MA, USA).

The ESSE-Ivanovo sample was sequenced using a target panel design that included 242 genes (Table S1) and about 2000 clinically relevant variants [11]. The ESSE-Vologda sample was sequenced using a target panel design that included 217 genes (Table S1) and about 18,000 variants. Genes and variants included in the panels are associated with diseases such as coronary artery disease, hypertension, stroke, obesity, diabetes mellitus, and osteoporosis.

The DNA libraries for the NGS custom panel were prepared using the SeqCap EZ Prime Choice Library kit (Roche, Basel, Switzerland). The sequencing was performed on a Nextseq 550 instrument (Illumina, San Diego, CA, USA) [11]. All sequencing stages were performed according to the manufacturers’ protocols.

### 2.5. Bioinformatic Analysis

The reads were aligned to the hg38 human genome with bwa mem [44] using the GATK v. 3.8 [45] custom pipeline [11]. Only variants passing GATK hard filtering and with a call rate above 90% were used. The ESSE-Ivanovo and ESSE-Vologda samples were not merged for further analysis due to differences in the target panel designs. We used PLINK v. 1.90 [46] to obtain identity-by-descent proportion estimates (PI_HAT = P(IBD=2) + P(IBD=1)/2) for all pairs. To ensure the absence of close relatives in the datasets, a younger participant was removed from each pair with PI_HAT > 0.33. Relatedness analysis by PLINK identified 173 and 13 individuals to be removed from ESSE-Ivanovo and ESSE-Vologda, respectively. Subsequently, PCA was conducted on individual genotypes using the Hail library v.0.2.83-b3151b4c4271. Outliers were identified through the implementation of hierarchical clustering using SciPy v. 1.7.3. PCA outlier analysis allowed us to remove four individuals from ESSE-Ivanovo and 15 individuals from ESSE-Vologda. Eight individuals from ESSE-Ivanovo and five from ESSE-Vologda were removed after checking covariate and lipid phenotypical data availability. The final ESSE-Ivanovo and ESSE-Vologda samples used in subsequent analyses consisted of 1673 and 817 individuals, respectively.

PRS data were collected from three relevant sources. Harmonized full summary statistics for Willer et al. [47] were downloaded from the GWAS Catalog [48] [GCST002223, GCST002222, GCST002221, GCST002216]. Significantly associated variants were retrieved from the original publication. In the case of Selvaraj et al. [49], only the significantly associated variants were available directly in the publication. In Xu et al. [50], both the PRS and GWAS summary statistics were available from the website. We performed the liftover of the variants from Xu et al. [50] to the GRCh38 human genome assembly with UCSC liftOver v. 1 [51].

PRS evaluation with respect to our population samples was further performed in three different ways:

1 PRS based on the classical approach with original effect sizes (referred to as the “classical method” for brevity) were evaluated using an in-house script that calculates PRS as a sum of risk allele dosages weighted by the risk allele effect estimates. The latter were obtained either from a PRS [50] or from a list of lead variants from GWAS [47,49]. All lead variants from Willer et al. [47] were included in the original target panel designs for ESSE-Ivanovo and ESSE-Vologda. It should be noted that, unlike those from Willer et al. [47], the original target panel designs used for the ESSE-Ivanovo and ESSE-Vologda samples included only a fraction of variants from PRS reported in Xu et al. [50] and significant GWAS hits from Selvaraj et al. [49] (Table 1).
2 For the two studies where summary statistics were available, we used the C+T (clumping and thresholding) approach provided by PRSice-2 v. 2.3.5 [52]. To identify the best clumping r^2^ (squared correlation coefficient) and *p*-value thresholds, we ran the software for r^2^ equal to 0, 0.1, 0.2, 0.3, 0.4, and 0.5. The *p*-value thresholds for individual variants ranged from 1 × 10^−100^ to 1 × 10^−4^ with a 5 × 10^−50^ uniform step. Both ESSE-Ivanovo and ESSE-Vologda were randomly split 4:1 for PRS development and further validation.
3 Finally, we constructed PRS using LDpred2 provided in bigsnpr v. 1.12.2 package [53] with the LDpred2-auto model with the HapMap3+ reference LD panel [54] to calculate the PRS. LDpred2 infers the posterior mean effect size of each risk allele by using information from the LD reference panel and the prior risk allele effect size distribution. This approach does not require an independent validation dataset, as the method estimates its hyperparameters directly from the GWAS summary statistics.

For PRSice-2 and LDpred2 PRS development, data from Selvaraj et al. [49] were not used, as this paper did not provide the complete summary statistics.

### 2.6. Statistical Analysis

PRS statistical analysis was performed with R v. 4.1.2 [55]. The phenotypical data distributions do not follow a normal distribution (Shapiro–Wilk test’s *p* < 0.05), although the shapes of the distributions have bell-like forms (Figure S1). Continuous variables were compared with the Mann–Whitney Wilcoxon test, and nominal variables were compared with the chi-square test. Continuous variables were presented as median and interquartile range or as mean and standard deviation. PRS values were Z-score normalized for subsequent analysis. To evaluate PRS, we constructed linear models for each of the lipid phenotypes, including the above-mentioned covariates: sex, age, smoking status, BMI, and statin intake. For blood lipid phenotypes, statin intake data for the moment of blood lipid measurement was used. For artery ultrasound parameters, statin intake data at the moment of ultrasound was used. The incremental R^2^ (coefficient of determination, percent of variance explained) was calculated for all PRS as the increase in R^2^ caused by the addition of PRS to the initial covariate-only model. For R^2^ values, confidence intervals were calculated using the boot v. 1.3-28 package. Spearman’s correlation coefficient estimates (ρ) and confidence intervals were acquired using the RVAideMemoire v. 0.9-83-7 package for each PRS. The associations between PRS and all phenotypes were evaluated using both R^2^ and ρ. PRS *p*-values were obtained by comparing linear models with and without PRS with ANOVA. Bonferroni correction was used to adjust for type 1 errors in cases where multiple testing was performed. Bonferroni correction is the most conservative method, which is widely used and does not require any assumption about the independence of the hypothesis tested.

## 3. Results

### 3.1. Sample Description

SSE-RF [35] from the Ivanovo (ESSE-Ivanovo) and Vologda (ESSE-Vologda) regions are provided in Table 2. In particular, ESSE-Vologda participants were found to be younger on average and have lower BMI, HDL-C, LDL-C, TC, and logTG levels.

The ATHEROGEN-Ivanovo substudy ultrasound data were available for 1028 ESSE-Ivanovo participants (61.4% of the complete sample), as detailed below. This percentage of participants is mostly explained by the age limitations of the ATHEROGEN-Ivanovo (see Section 2). Out of 1028 ATHEROGEN-Ivanovo participants, 288 were male (28.0%). The median age of the subsample was 53 [47, 59]. Ultrasound parameters of atherosclerosis were found to differ between carotid and femoral arteries (Table 3, Figure S2).

### 3.2. PRS Based on the Classical Method Evaluation

PRS based on the classical method evaluation required extraction of reported significant GWAS variants for Selvaraj et al. and Willer et al. [47,49] and some additional processing of PRS in the case of Xu et al. [50].

Table 4 presents the resulting R^2^ estimates: from 3.75% to 6.41% for HDL-C, 4.54% to 6.23% for LDL-C, 2.74% to 5.98% for TC, and 0.42% to 3.57% for logTG in the case of ESSE-Ivanovo. The corresponding R^2^ estimates for ESSE-Vologda ranged from 4.13% to 5.25% for HDL-C, 4.60% to 9.61% for LDL-C, 4.39% to 6.44% for TC, and 1.58% to 5.77% for logTG (Table 4). The highest phenotype value increase per unit of standard deviation (1 PRS SD) was observed in the LDL-C PRS for the ESSE-Vologda Selvaraj et al. [49]: 0.30 [95% CI, 0.25–0.37] mmol/L; the lowest in the logTG for ESSE-Ivanovo Xu et al. [50]: 0.03 [95% CI, 0.01–0.06] (Table S2). The mean lipid values were observed to increase in accordance with the strata of all analyzed PRS (Figures S3 and S4).

Generally, PRS based on the classical method displayed the best performance in the case of Willer et al. [47], followed by Selvaraj et al. [49]. The PRS based on Xu et al. [50] performed the worst, which may be explained by the lack of variants from this PRS in our sequencing data (see Table 1 in Section 2).

### 3.3. PRSice-2 PRS Development and Evaluation

**Figure 1:**
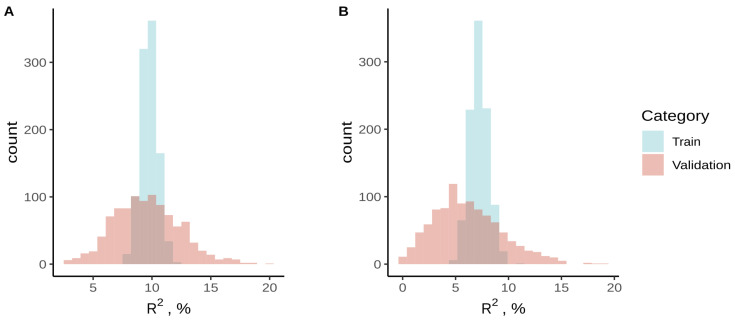
Comparison of R^2^ distributions between training and validation data for (**A**) ESSE-Ivanovo HDL-C phenotype and (**B**) ESSE-Vologda logTG phenotype. No significant difference of means was shown for the ESSE-Ivanovo HDL-C phenotype, and a significant difference was shown for the ESSE-Vologda logTG phenotype.

To assess the feasibility of PRSice-2 for developing polygenic risk scores (PRS) in small sample sets, we initially investigated the potential for overfitting in PRS models. The ESSE-Ivanovo and ESSE-Vologda samples were randomly split 1000 times into training and validation sets with a ratio of 4:1. Data from Willer et al. [47] were used to fit the PRS at an r^2^ equal to 0.2, with a lower *p*-value threshold of 1 × $10^{-100}$ and an upper threshold of 1 × $10^{-4}$ with a uniform 5 × $10^{-50}$ step. The PRS were initially fitted on the training data, and subsequently, the R^2^ estimate was obtained on the validation data for best-fit PRS parameters. The paired *t*-test was used to compare the average values of R^2^ in training and validation data and confirmed that in the case of ESSE-Ivanovo, there were no significant differences (*p* > 0.05) for all lipid phenotypes and no difference for the TC phenotype in the case of ESSE-Vologda (Figure 1). There were significant differences in HDL-C, LDL-C, and logTG phenotypes in the latter case, however. This result might indicate the possible overfitting of PRS by PRSice-2 for these three phenotypes in ESSE-Vologda, most likely caused by the small size of this sample.

PRS development with PRSice-2 required an independent validation dataset. We randomly split the ESSE-Ivanovo and ESSE-Vologda samples in a 4:1 ratio to form the training and validation datasets. This resulted in the ESSE-Ivanovo training dataset of 1338 individuals and the validation dataset of 335 individuals, and 654 and 163 individuals in the case of ESSE-Vologda, respectively. Only subsets of variants for which the full summary statistics were available in Willer et al. [47] and Xu et al. [50] matched sequenced variants in ESSE-Ivanovo and ESSE-Vologda (Table S3).

Overall, the developed PRS for ESSE-Ivanovo included from 62 variants (Xu et al. [50], logTG) to 260 variants (Willer et al. [47], TC); the developed PRS for ESSE-Vologda included from 59 variants (Xu et al. [50], HDL-C) to 361 variants (Willer et al. [47], TC). All PRS developed with PRSice-2 demonstrated almost equal or higher R^2^ estimates than the PRS based on the classical method (Tables S4 and S5). In most cases, R^2^ estimates between training and validation samples did not differ remarkably (Table 5), with the exception of logTG for the ESSE-Vologda that showed clear signs of overfitting: results on the validation dataset were significantly worse than on the training dataset, and the addition of the PRS to the covariate-only model turned out to be non-significant (*p* > 0.05, Table S5).

All other PRS produced significant results, with the lowest R^2^ estimates observed for logTG phenotype in ESSE-Ivanovo: R^2^ = 3.03% [95% CI, 0.70–7.14%] for logTG PRS in Xu et al.’s data [50] on the validation sample, and the highest estimate: R^2^ = 13.20% [95% CI, 4.68–25.09%] for LDL-C in Willer et al.’s data [47] on the validation sample. The highest 1 PRS SD increase for validation was shown for Willer et al.’s data [47] for LDL-C for the ESSE-Vologda sample: 0.38 [95% CI, 0.24–0.52] mmol/L. The lowest value, in turn, was observed for Willer et al.’s data [47] of the HDL-C score in the case of ESSE-Ivanovo: 0.08 [95% CI, 0.05–0.11]. Mean lipid values were shown to increase with strata of all PRS (Figures S5 and S6), although some fluctuations were observed for TC and logTG PRS in the ESSE-Vologda data. Both GWAS summary statistics used for PRS development displayed approximately equally good performance.

### 3.4. LDpred2 PRS Development and Evaluation

The final set of PRS was developed using LDpred2 [53]. The analysis had to be limited to the HapMap3+ variants, since the method relies on this LD reference set provided by the authors [54]. This restricted the analysis to 3318–3342 (depending on lipid phenotype) variants from Willer et al. [47] and 4425 variants from Xu et al. [50] for the ESSE-Ivanovo sample, and 12,836–12,864 variants from Willer et al. [47] and 17,244 variants from Xu et al. [50] for the ESSE-Vologda sample (Table S3).

**Figure 2:**
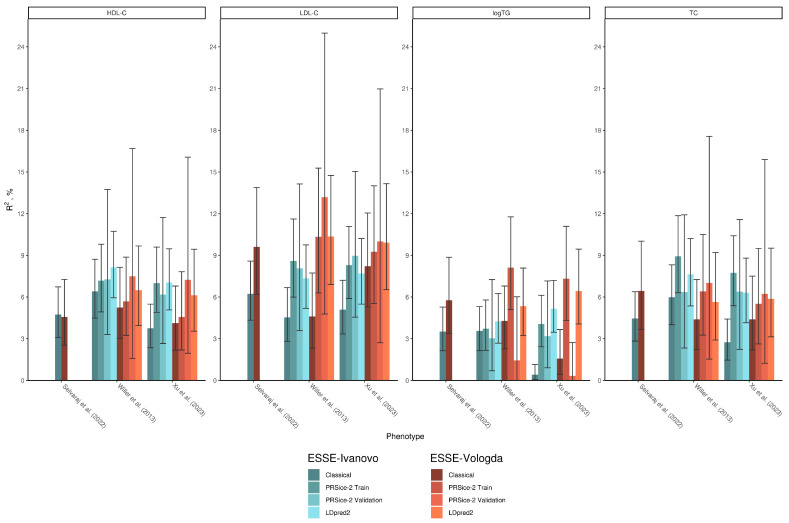
Comparison of R^2^ estimates across all developed PRS scales. For PRSice-2, R^2^ estimates from both training and validation datasets are given. Estimates are provided along with 95% CI. HDL-C—high-density lipoprotein cholesterol, LDL-C—low-density lipoprotein cholesterol, TC—total cholesterol, logTG—logarithmic triglycerides.

LDpred2, in some cases, has shown slightly worse results for R^2^ and ρ estimates compared to PRSice-2. However, it consistently outperformed PRS based on the classical method estimates (Table 6 and Table S6, Figure 2 and Figure S7). The lowest 1 PRS SD increase was shown for Xu et al.’s data [50] on HDL-C: 0.08 mmol/L [95% CI, 0.06–0.10]. In contrast, the highest increase was observed for the Xu et al. data [50] on TC: 0.33 mmol/L [95% CI, 0.28–0.38]. Mean phenotypical values were shown to increase with strata of all PRS analyzed (Figures S8 and S9).

### 3.5. Clinical Utility of PRS for Atherosclerosis

To evaluate the clinical applicability of developed PRS scores, we tested the degree of association between 28 PRS scores developed for ESSE-Ivanovo (7 scores: 3 based on the classical method, 2 developed with PRSice-2, and 2 with LDpred2, each for 4 lipid phenotypes) and 12 ultrasound parameters of carotid and femoral atherosclerosis for each participant of ATHEROGEN-Ivanovo.

We excluded blood lipid PRS that did not show significant associations with ultrasound parameters (Bonferroni corrected *p* > 0.05/28 = 1.78 × $10^{-3}$) from subsequent analysis. This resulted in 73 significant associations with atherosclerotic phenotypes with blood lipid PRS for all phenotypes (Table S7): 22 for PRS based on the classical method, 17 for PRS developed using PRSice-2, and 34 for PRS constructed with LDpred2. No significant associations have been shown for IMT in the right carotid and femoral arteries.

The highest R^2^ was achieved by TC PRS based on Willer et al. [47] developed with PRSice-2 in relation to maximum stenosis of carotid arteries: 1.83% [95% CI, 0.28–2.48]. The lowest R^2^ was observed for HDL-C based on Willer et al. [47] developed with LDpred2 and plaque number in femoral arteries: 0.73% [95% CI, 0.10–1.86]. In this case, the ρ estimate was negative: −0.08 ([95% CI, −0.014–−0.02], nominally significant with *p* = 8.73 × $10^{-3}$). Some of the ρ estimates turned out to be insignificant with a *p* > 1.78 × $10^{-3}$: 26 PRS out of 73 with significant associations with atherosclerotic phenotypes (35.6%) did not show significant correlations. The average R^2^ across 73 PRS scales equaled 0.94%.

## 4. Discussion

This study investigates the usability of currently available blood lipid GWAS results [47,49,50] for the development of PRS for the samples from the European part of the Russian Federation: ESSE-Vologda and ESSE-Ivanovo. Several approaches to PRS application have been explored, including the use of PRS based on the classical method with original variant effect sizes and the development of PRS with PRSice-2 and LDpred2.

As expected, the PRS based on the classical method demonstrated the poorest performance, potentially due to the inability to account for genetic differences between base and target populations. All data used for the PRS development originated either from multiethnic [49] or European populations [47,50]. Although the Ivanovo and the Vologda regions belong to the European part of Russia, it has been shown that even in the demographically similar regions of the country, some admixture of Finnish and Asian ancestry is typical [12]. PRS developed by PRSice-2 and LDpred2 performed better than the classical method for the majority of phenotypes, except for the logTG PRS in ESSE-Vologda, which is a clear case of overfitting due to the small sample size. While neither method is unequivocally superior, we recommend the LDpred2-auto model for smaller samples, as it requires no validation set and has the HapMap3+ LD panel provided by the authors [54]. In most cases, it also performs slightly better than PRSice-2.

Our approach to PRS development with PRSice-2 and LDpred2 allowed us to outperform the previous results acquired for the Russian population [56], which achieved an R^2^ roughly equal to 5% for TG and TC, and an R^2^ equal to 6% for HDL. We achieved an increase of the incremental R^2^ by 2-4% depending on the approach and the phenotype investigated. In general, the R^2^ estimates of the PRS are close to the estimates described for other populations [15,57,58].

We took this a step further and evaluated the clinical utility of blood lipid PRS for predicting carotid and femoral atherosclerosis. For this purpose, we assessed the association of four lipid phenotype PRS with ultrasound parameters of atherosclerosis. The availability of GWAS data for these parameters remains limited [59,60]; thus, the application of PRS from related phenotypes may be advantageous. The maximal R^2^ equaled 1.83% (TC PRS based on Willer et al. [47], developed with PRSice-2 in relation to maximum stenosis of carotid arteries), and the maximal ρ was equal to 0.13 (LDL-C PRS based on the classical method based on Willer et al. [47] in relation to plaque score of carotid arteries, *p* = 3.32 × $10^{-5}$). More associations (40 out of 73, roughly 55%) have been identified for femoral arteries. Associations have been shown for all four blood lipid phenotype PRS. It is important to emphasize that we demonstrated the inverse association between Willer et al.’s data [47] on HDL-C PRS developed with LDpred2 and plaque number in femoral arteries, with a negative ρ value of −0.08 (*p* = 8.71 × $10^{3}$). PRS for other blood lipids was positively correlated with carotid and femoral atherosclerosis, with the best association results obtained for LDL-C and TC PRS. These observations are consistent with the general knowledge that TC, LDL-C, and TG are major risk factors for atherosclerotic cardiovascular disease [61]. In the case of HDL-C, low levels are associated with increased risk of atherosclerosis [62], yet extremely high HDL-C levels are known to be associated with all-cause mortality [63]. As we observed almost no high-level HDL-C individuals, the observation of inverse associations reflects the literature data. The study showed no significant associations for IMT in the right carotid and femoral arteries, which is in line with recommendation not to use routine IMT measurement in risk assessment and prediction of cardiovascular events [64]. Our findings suggest the potential for further utilization of blood lipid PRS in predicting peripheral atherosclerosis risk. Future studies exploring the GWAS for atherosclerosis ultrasound parameters would greatly benefit the possibility of clinical application of atherosclerosis-related PRS.

This study also had several limitations. Firstly, the study included fewer samples than typically used for PRS development [4,16]. Secondly, the sequencing was performed using target panel design [11,36,37], thus omitting some variants that would possibly improve the performance of PRS evaluated and restricting the adjustment for population structure. The expansion of available whole genome sequencing data would further boost the performance of PRS.

## 5. Conclusions

The existing blood lipid PRS may display limited transferability to the Russian population. On the other hand, PRS fine-tuning using pre-existing GWAS results with PRSice-2 and LDpred2 improves the performance of blood lipid PRS, which may be used to further investigate the role of blood lipid-associated variants in atherosclerosis.

## Figures and Tables

**Table 1:** Variant statistics in each of the PRS based on the classical method. Variant counts for the ESSE-Ivanovo and the ESSE-Vologda samples are also provided as percent of the original variant set size.

Study	Phenotype	Variant Count in the Original Study	Variants in ESSE-Ivanovo	Variants in ESSE-Vologda
Willer et al. [47]	HDL-C	71	71 (100%)	71 (100%)
	LDL-C	57	57 (100%)	57 (100%)
	logTG	73	73 (100%)	73 (100%)
	TC	39	39 (100%)	39 (100%)
Selvaraj et al. [49]	HDL-C	357	63 (17.6%)	89 (24.9%)
	LDL-C	338	48 (14.2%)	67 (19.8%)
	logTG	289	38 (13.1%)	73 (25.3%)
	TC	324	51 (15.7%)	75 (23.2%)
Xu et al. [50]	HDL-C	223	40 (17.9%)	71 (31.8%)
	LDL-C	392	35 (8.9%)	57 (14.5%)
	logTG	339	41 (12.0%)	71 (20.8%)
	TC	329	34 (10.3%)	81 (24.6%)

HDL-C—high-density lipoprotein cholesterol; LDL-C—low-density lipoprotein cholesterol; TC—total cholesterol; logTG—logarithmic triglycerides.

**Table 2:** ESSE-Ivanovo and ESSE-Vologda covariate and phenotype data summary.

Parameters	ESSE-Ivanovo	ESSE-Vologda	*p*
Total number of participants	1673	817	
Age, years, Me [Q25, Q75]	50 [40, 57]	47 [35, 56]	<0.001
Males, *n (%)*	624 (37.4%)	346 (43.4%)	0.017
BMI, kg/m^2^, Me [Q25, Q75]	28.1 [24.8, 31.9]	26.2 [23.1, 30.3]	<0.001
Statins, *n (%)*	95 (5.7%)	20 (2.4%)	<0.001
Smoking status, *n (%)*	Never smoked: 1070 (63.9%) Ex-smokers: 302 (18.1%) Smoke: 301 (18.0%)	Never smoked: 461 (56.4%) Ex-smokers: 172 (21.1%) Smoke: 184 (22.5%)	0.001
HDL-C, mmol/L, Me [Q25, Q75]	1.38 [1.18, 1.62]	1.26 [1.06, 1.47]	<0.001
LDL-C, mmol/L, Me [Q25, Q75]	3.50 [2.73, 4.30]	3.36 [2.74, 4.04]	0.003
TC, mmol/L, Me [Q25, Q75]	5.65 [4.87, 6.45]	5.15 [4.47, 5.98]	<0.001
TG, mmol/L, Me [Q25, Q75]	1.23 [0.87, 1.85]	1.09 [0.76, 1.55]	<0.001

Median values and the 25th and 75th percentiles are provided for continuous variables. For nominal values, counts are provided. Differences between samples were compared with the chi-square test (sex, statin therapy, smoking status) or the Mann–Whitney Wilcoxon test (age, BMI, HDL-C, LDL-C, TC, logTG), with *p*-values reported in the table. BMI—body mass index; HDL-C—high-density lipoprotein cholesterol; LDL-C—low-density lipoprotein cholesterol; Me—median; TC—total cholesterol; logTG—logarithmic triglycerides.

**Table 3:** ATHEROGEN-Ivanovo ultrasound parameters of carotid and femoral atherosclerosis. The measurements included maximum and total stenosis, plaque number and plaque score, and intima-media thickness (IMT) on both sides.

Parameter	Carotid Arteries	Femoral Arteries	*p*
Maximum stenosis, %, Me [Q25, Q75]	24 [0, 31]	0 [0, 24]	<0.001
Total stenosis, %, Me [Q25, Q75]	26 [0, 68]	0 [0, 28]	<0.001
Plaque number, *n*, mean ± SD	1.51 ± 1.56	0.91 ± 1.54	<0.001
Plaque score, mm, mean ± SD	2.85 ± 3.39	2.32 ± 4.58	<0.001
IMT (right), mm, Me [Q25, Q75]	0.72 [0.63, 0.82]	0.62 [0.53, 0.81]	<0.001
IMT (left), mm, Me [Q25, Q75]	0.73 [0.64, 0.84]	0.61 [0.51, 0.80]	<0.001

Median values and the 25th and 75th percentiles, or mean and standard deviation are provided for each parameter. Carotid and femoral ultrasound parameters were compared with the Mann–Whitney Wilcoxon test, with *p*-values provided in the table. IMT—intima-media thickness; Me—median, SD—standard deviation.

**Table 4:** Estimates of R^2^ for PRS based on the classical method.

Study	Phenotype	Variant Count	R^2^, %	R^2^ 95% CI	*p*
ESSE-Ivanovo					
Selvaraj et al. [49]	HDL-C	63	4.74	[3.12–6.71]	1.53 × $10^{-23}$
	LDL-C	48	6.23	[4.31–8.56]	2.37 × $10^{-28}$
	logTG	38	3.51	[2.12–5.25]	4.71 × $10^{-18}$
	TC	51	4.45	[2.81–6.36]	3.27 × $10^{-21}$
Willer et al. [47]	HDL-C	71	6.41	[4.48–8.72]	1.43 × $10^{-31}$
	LDL-C	57	4.54	[2.81–6.68]	6.85 × $10^{-21}$
	logTG	39	3.57	[2.14–5.31]	2.33 × $10^{-18}$
	TC	73	5.98	[4.01–8.31]	3.40 × $10^{-28}$
Xu et al. [50]	HDL-C	40	3.75	[2.35–5.49]	7.78 × $10^{-19}$
	LDL-C	35	5.09	[3.34–7.21]	2.68 × $10^{-23}$
	logTG	41	0.42	[0.05–1.15]	3.18 × $10^{-03}$
	TC	34	2.74	[1.46–4.41]	1.53 × $10^{-13}$
ESSE-Vologda					
Selvaraj et al. [49]	HDL-C	89	4.57	[2.50–7.24]	1.08 × $10^{-11}$
	LDL-C	67	9.61	[6.17–13.85]	2.31 × $10^{-22}$
	logTG	73	5.77	[3.36–8.84]	1.48 × $10^{-15}$
	TC	75	6.44	[3.65–9.99]	4.19 × $10^{-15}$
Willer et al. [47]	HDL-C	71	5.25	[3.02–8.14]	3.00 × $10^{-13}$
	LDL-C	57	4.60	[2.32–7.72]	3.38 × $10^{-11}$
	logTG	39	4.28	[2.28–6.79]	8.41 × $10^{-12}$
	TC	73	4.40	[2.22–7.25]	1.13 × $10^{-10}$
Xu et al. [50]	HDL-C	71	4.13	[2.17–6.79]	1.15 × $10^{-10}$
	LDL-C	57	8.22	[5.28–12.05]	3.43 × $10^{-19}$
	logTG	81	1.58	[0.44–3.66]	3.89 × $10^{-5}$
	TC	71	4.39	[2.19–7.51]	1.16 × $10^{-10}$

Each R^2^ estimate is provided as the percent of variance explained, the 95% CI, and the *p*-value from the ANOVA to measure the significance of the addition of PRS to the model. CI—confidence interval; HDL-C—high-density lipoprotein cholesterol; LDL-C—low-density lipoprotein cholesterol; TC—total cholesterol; logTG—logarithmic triglycerides.

**Table 5:** Comparison of R^2^ estimates in training and validation samples in the ESSE-Ivanovo and ESSE-Vologda for PRS developed with PRSice-2.

Study	Phenotype	Variant Count	R^2^ Training, %	R^2^ Validation, %
ESSE-Ivanovo				
Willer et al. [47]	HDL-C	129	7.18	7.27
	LDL-C	139	8.60	8.07
	logTG	199	3.72	3.03
	TC	260	8.94	6.36
Xu et al. [50]	HDL-C	89	7.01	6.18
	LDL-C	98	8.30	8.97
	logTG	62	4.06	3.19
	TC	98	7.74	6.39
ESSE-Vologda				
Willer et al. [47]	HDL-C	240	5.69	7.49
	LDL-C	322	10.33	13.20
	logTG	361	8.11	1.44
	TC	333	6.41	7.02
Xu et al. [50]	HDL-C	59	4.56	7.24
	LDL-C	173	9.26	10.00
	logTG	146	7.32	0.33
	TC	191	5.52	6.22

Each R^2^ estimate is the percent of variance in training or validation set. HDL-C—high-density lipoprotein cholesterol; LDL-C—low-density lipoprotein cholesterol; TC—total cholesterol; logTG—logarithmic triglycerides.

**Table 6:** Comparison of R^2^ estimates in the ESSE-Ivanovo and ESSE-Vologda samples for PRS developed with LDpred2.

Study	Phenotype	R^2^, %	95% CI	*p*
ESSE-Ivanovo				
Willer et al. [47]	HDL-C	7.18	[5.94–10.82]	4.59 × $10^{-40}$
	LDL-C	8.60	[5.20–9.82]	2.65 × $10^{-33}$
	logTG	3.72	[2.72–6.26]	1.29 × $10^{-21}$
	TC	8.94	[5.40–10.25]	7.52 × $10^{-36}$
Xu et al. [50]	HDL-C	7.01	[5.06–9.43]	1.15 × $10^{-34}$
	LDL-C	8.30	[5.55–10.2]	6.75 × $10^{-35}$
	logTG	4.06	[3.43–7.24]	4.85 × $10^{-26}$
	TC	7.74	[4.15–8.67]	1.04 × $10^{-29}$
ESSE-Vologda				
Willer et al. [47]	HDL-C	5.69	[3.94–9.67]	3.73 × $10^{-16}$
	LDL-C	10.33	[6.90–14.76]	4.65 × $10^{-24}$
	logTG	8.11	[3.22–8.08]	1.74 × $10^{-14}$
	TC	6.41	[2.91–9.21]	2.24 × $10^{-13}$
Xu et al. [50]	HDL-C	4.56	[3.55–9.43]	2.67 × $10^{-15}$
	LDL-C	9.26	[6.53–14.15]	4.67 × $10^{-23}$
	logTG	7.32	[4.06–9.45]	3.11 × $10^{-17}$
	TC	5.52	[3.14–9.52]	7.10 × $10^{-14}$

Each R^2^ estimate is provided as the percent of variance explained, the 95% CI, and the *p*-value from the ANOVA to measure the significance of the addition of PRS to the model. CI—confidence interval; HDL-C—high-density lipoprotein cholesterol; LDL-C—low-density lipoprotein cholesterol; TC—total cholesterol; logTG—logarithmic triglycerides.

## Data Availability

The data used and/or analyzed during the current study are available from the corresponding authors on reasonable request. Individual genotype information cannot be made available in order to protect participant privacy.

## Supplementary Materials

The following supporting information can be downloaded at: https://www.mdpi.com/article/10.3390/biomedicines12122798/s1, Figure S1: Distributions of phenotypes (LDL-C, HDL-C, TC, and logTG) in ESSE-Ivanovo and ESSE-Vologda; Figure S2: Distributions of ATHEROGEN-Ivanovo phenotypes; Figure S3: Distribution of mean lipid phenotype values by PRS based on classical method strata in ESSE-Ivanovo; Figure S4: Distribution of mean lipid phenotype values by PRS based on classical method strata in ESSE-Vologda; Figure S5: Distribution of mean lipid phenotype values by PRSice-2 PRS strata in ESSE-Ivanovo (train and validation dataset combined); Figure S6: Distribution of mean lipid phenotype values by PRSice-2 PRS strata in ESSE-Vologda (train and validation dataset combined); Figure S7: Comparison of Spearman’s rho estimates across all developed scales; Figure S8: Distribution of mean lipid phenotype values by LDpred2 PRS strata in ESSE-Ivanovo; Figure S9: Distribution of mean lipid phenotype values by LDpred2 PRS strata in ESSE-Vologda; Table S1: List of genes included in the ESSE-Ivanovo and ESSE-Vologda target panels; Tabel S2: PRS based on classical method results for the ESSE-Ivanovo and ESSE-Vologda samples; Table S3: Variant counts for each sample/source/phenotype combination after variant intersection with sequencing results; Table S4: PRS results for scores, developped with PRSice-2 on the ESSE-Ivanovo sample; Table S5: PRS results for scores, developed with PRSice-2 on the ESSE-Vologda sample; Table S6: Results for PRS developed with LDpred2; Table S7: Atherosclerotic parameter prediction using blood lopid scores.

## Abbreviations

The following abbreviations are used in this manuscript:

BMI	body mass index
CCA	common carotid arteries
CFA	common femoral arteries
ESSE-RF	Epidemiology of Cardiovascular Diseases and Risk Factors in Regions of the Russian Federation
GWAS	genome-wide association studies
HDL-C	high-density lipoprotein cholesterol
ICA	internal carotid arteries
IMT	intima-media thickness
LDL-C	low-density lipoprotein cholesterol
NGS	next-generation sequencing
SD	standard deviation
SFA	superficial femoral arteries
TC	total cholesterol
TG	triglycerides
