# Supplementary material for: Blood Lipid Polygenic Risk Score Development and Application for Atherosclerosis Ultrasound Parameters
Source: Biomedicines. 2024 Dec 10;12(12):2798. doi: 10.3390/biomedicines12122798 (PMC11673070; doi:10.3390/biomedicines12122798)
Supplement: Supplementary file 1 [file biomedicines-12-02798-s001.zip › Supplementary_figures.pdf]

Figure S1

Distributions of phenotypes (LDL-C, HDL-C, TC, and logTG) in ESSE-Ivanovo and ESSE-Vologda. A) HDL-C (mmol/L) distributions in ESSE-Ivanovo and ESSE-Vologda. B) LDL-C (mmol/L) distribution in ESSE-Ivanovo and ESSE-Vologda. C) TC (mmol/L) distribution in ESSE-Ivanovo and ESSE-Vologda. D) logTG distributions in ESSE-Ivanovo and ESSE-Vologda. HDL-C — high-density lipoprotein cholesterol; LDL-C — low-density lipoprotein cholestero;, TC — total cholesterol; logTG — logarithmic triglycerides.

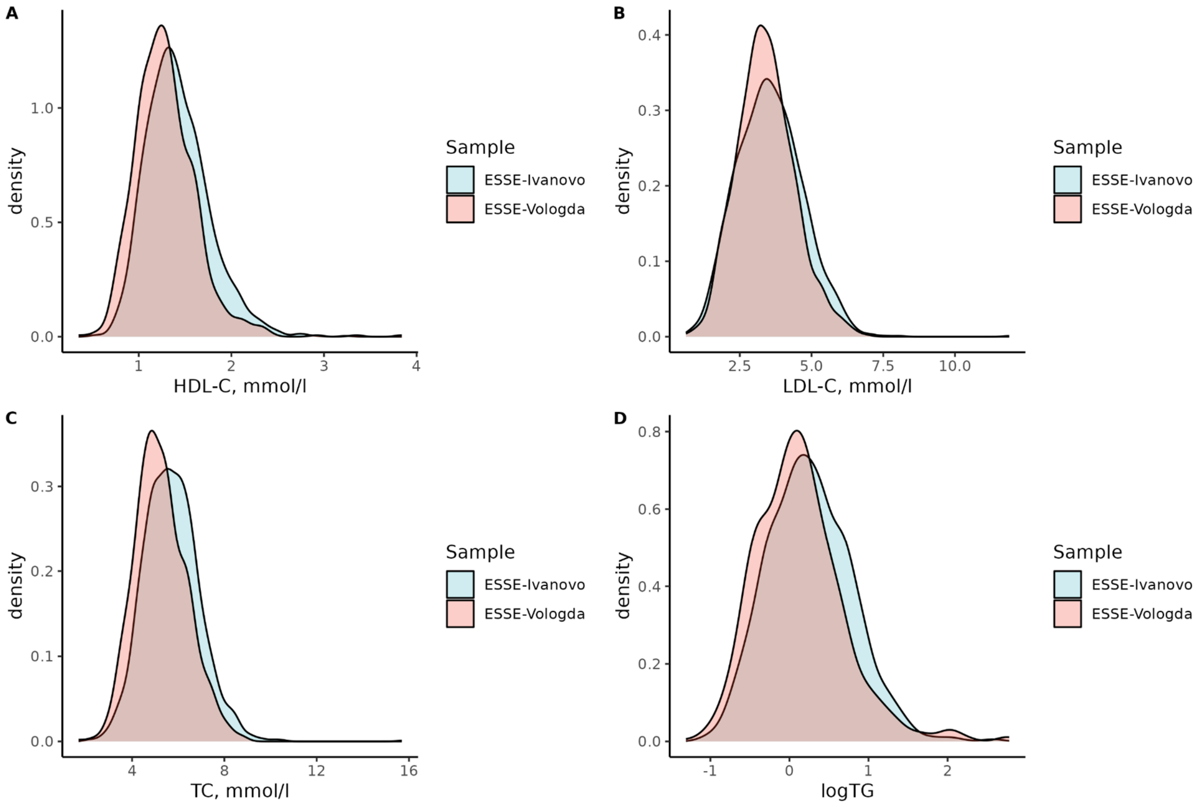

Figure S2

Distributions of ATHEROGEN-Ivanovo phenotypes are plotted as histograms. A) Distribution of maximum stenosis percent in carotid and femoral arteries. B) Distribution of total stenosis percent in carotid and femoral arteries. C) Distribution of plaque number in carotid and femoral arteries. D) Distribution of plaque score (maximum thickness of all plaques) in carotid and femoral arteries. E) Distribution of intima-media thickness (IMT) in the carotid and femoral arteries on the left. F) Distribution of intima-media thickness (IMT) in the carotid and femoral arteries on the right.

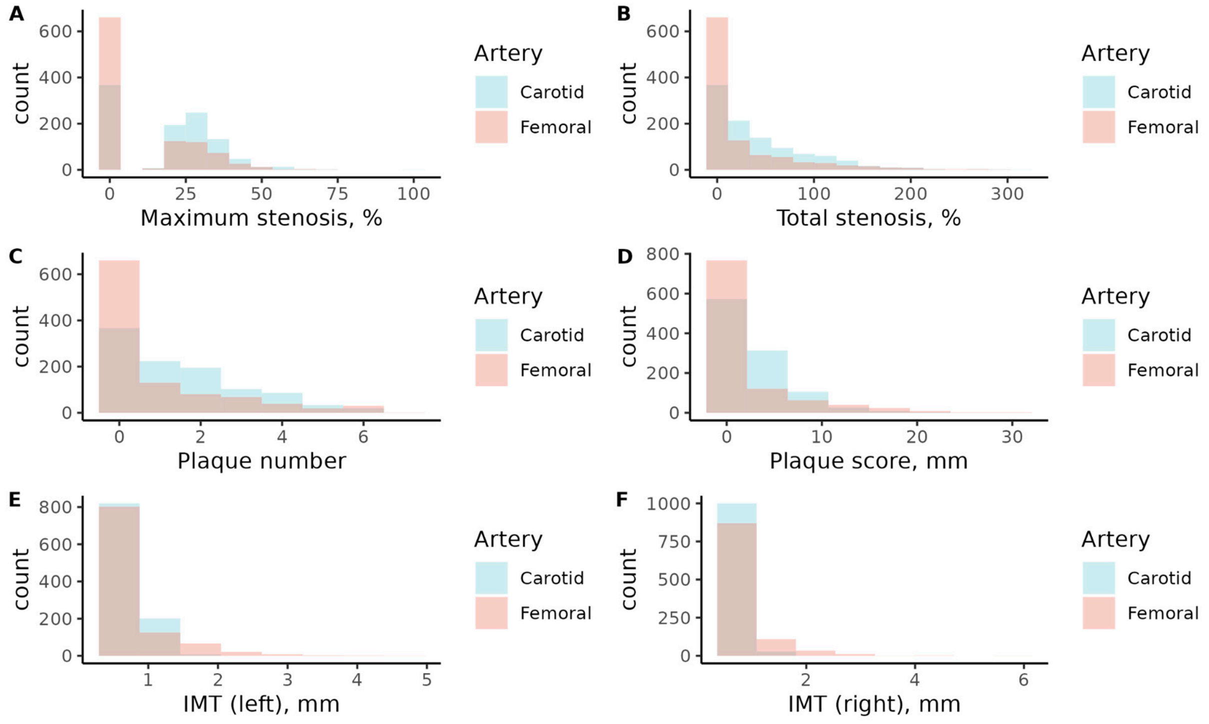

Figure S3

Distribution of mean lipid phenotype values by PRS based on classical method strata in ESSE-Ivanovo. Score values were calculated as the sum of risk allele dosage values (0, 1, 2) weighted with allele effect estimates. The variants were either provided directly by the authors [1], or as a list of significant hits in GWAS summary statistics [2,3]. Strata were calculated by sorting PRS z-scores from lowest to highest and splitting them into 10 equal parts. Scores A-D are from Selvaraj et al. [3], scores E-H are from Willer et al [2], scores I-L are from Xu et al. [1]. Scores A, E, I display the distribution of mean HDL-C levels (with 95% CI) in mmol/L across 10 PRS strata; scores B, F, J display the distribution of mean LDL-C levels (with 95% CI) in mmol/L across 10 PRS strata; scores C, G, K display distribution of mean TC levels (with 95% CI) in mmol/L across 10 PRS strata; scores D, H, L display the distribution of mean logTG levels (with 95% CI) across 10 PRS strata. HDL-C — high-density lipoprotein cholesterol; LDL-C — low-density lipoprotein cholestero;, TC — total cholesterol; logTG — logarithmic triglycerides.

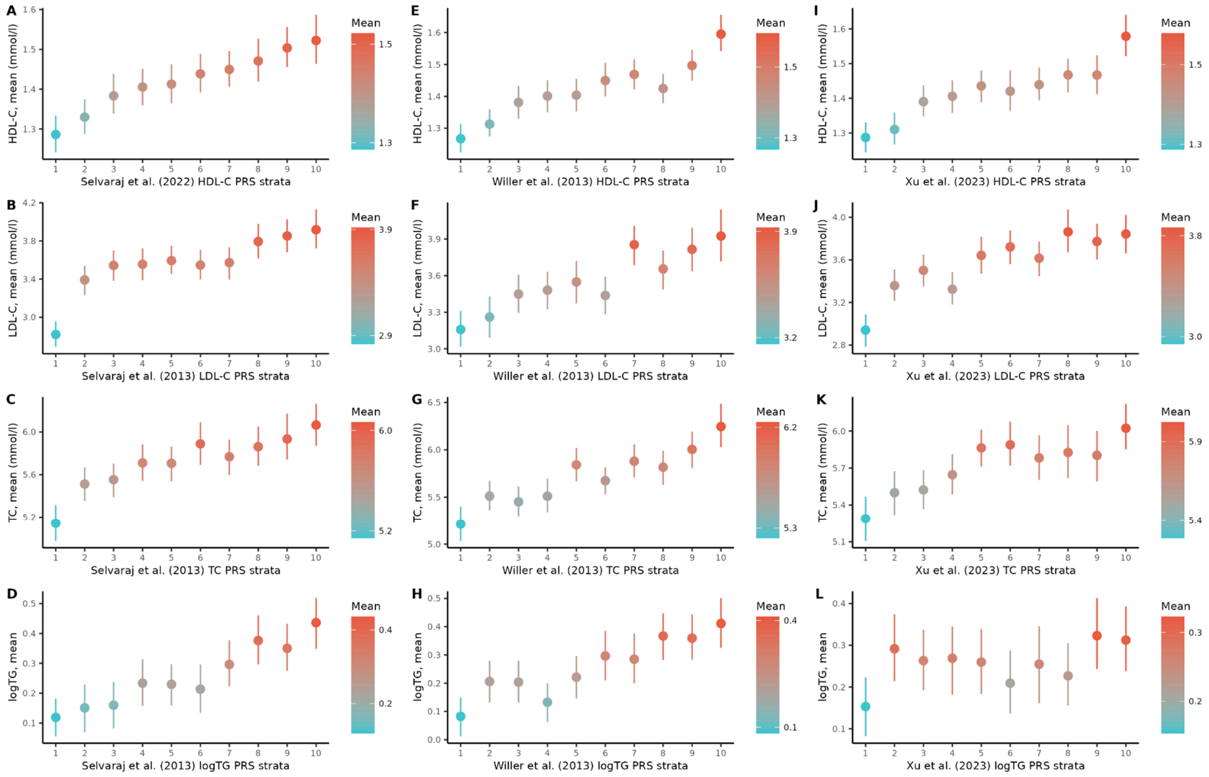

Figure S4

Distribution of mean lipid phenotype values by PRS based on classical method strata in ESSE-Vologda. Score values were calculated as the sum of risk allele dosage values (0, 1, 2) weighted with allele effect estimates. The variants were either provided directly by the authors [1], or as a list of significant hits in GWAS summary statistics [2,3]. Strata were calculated by sorting PRS z-scores from lowest to highest and splitting them into 10 equal parts. Scores A-D are from Selvaraj et al. [3], scores E-H are from Willer et al [2], scores I-L are from Xu et al. [1]. Scores A, E, I display the distribution of mean HDL-C levels (with 95% CI) in mmol/L across 10 PRS strata; scores B, F, J display the distribution of mean LDL-C levels (with 95% CI) in mmol/L across 10 PRS strata; scores C, G, K display distribution of mean TC levels (with a 95% CI) in mmol/L across 10 PRS strata; scores D, H, L display distribution of mean logTG levels (with 95% CI) across 10 PRS strata. HDL-C — high-density lipoprotein cholesterol; LDL-C — low-density lipoprotein cholestero;, TC — total cholesterol; logTG — logarithmic triglycerides.

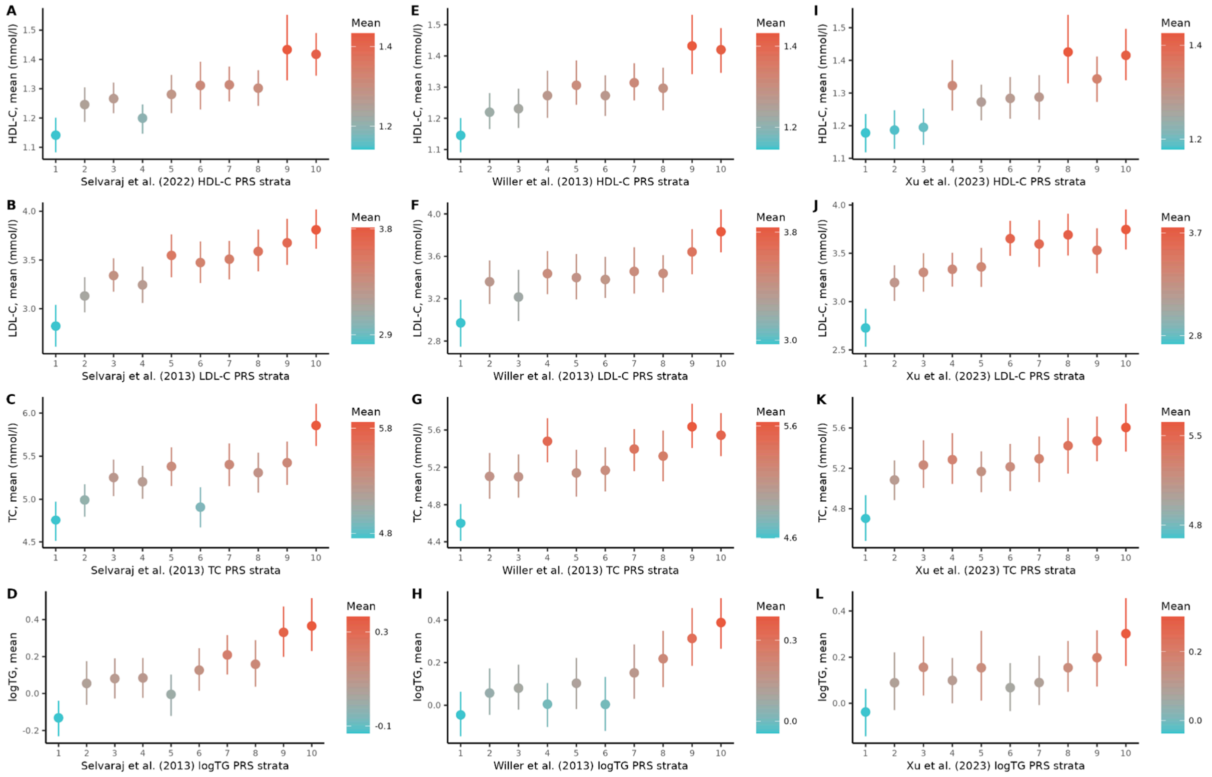

Figure S5

Distribution of mean lipid phenotype values by PRSice-2 PRS strata in ESSE-Ivanovo (train and validation dataset combined). Strata were calculated by sorting PRS z-scores from lowest to highest and splitting them into 10 equal parts. Scores A-D are from Willer et al. [2], scores E-H are from Xu et al. [1]. Scores A and E display the distribution of mean HDL-C levels (with 95% CI) in mmol/L across 10 PRS strata; scores B and F display the distribution of mean LDL-C levels (with 95% CI) in mmol/L across 10 PRS strata; scores C and G display the distribution of mean TC levels (with 95% CI) in mmol/L across 10 PRS strata; scores D and H display the distribution of mean logTG levels (with 95% CI) across 10 PRS strata. HDL-C — high-density lipoprotein cholesterol; LDL-C — low-density lipoprotein cholesterol;, TC — total cholesterol; logTG — logarithmic triglycerides.

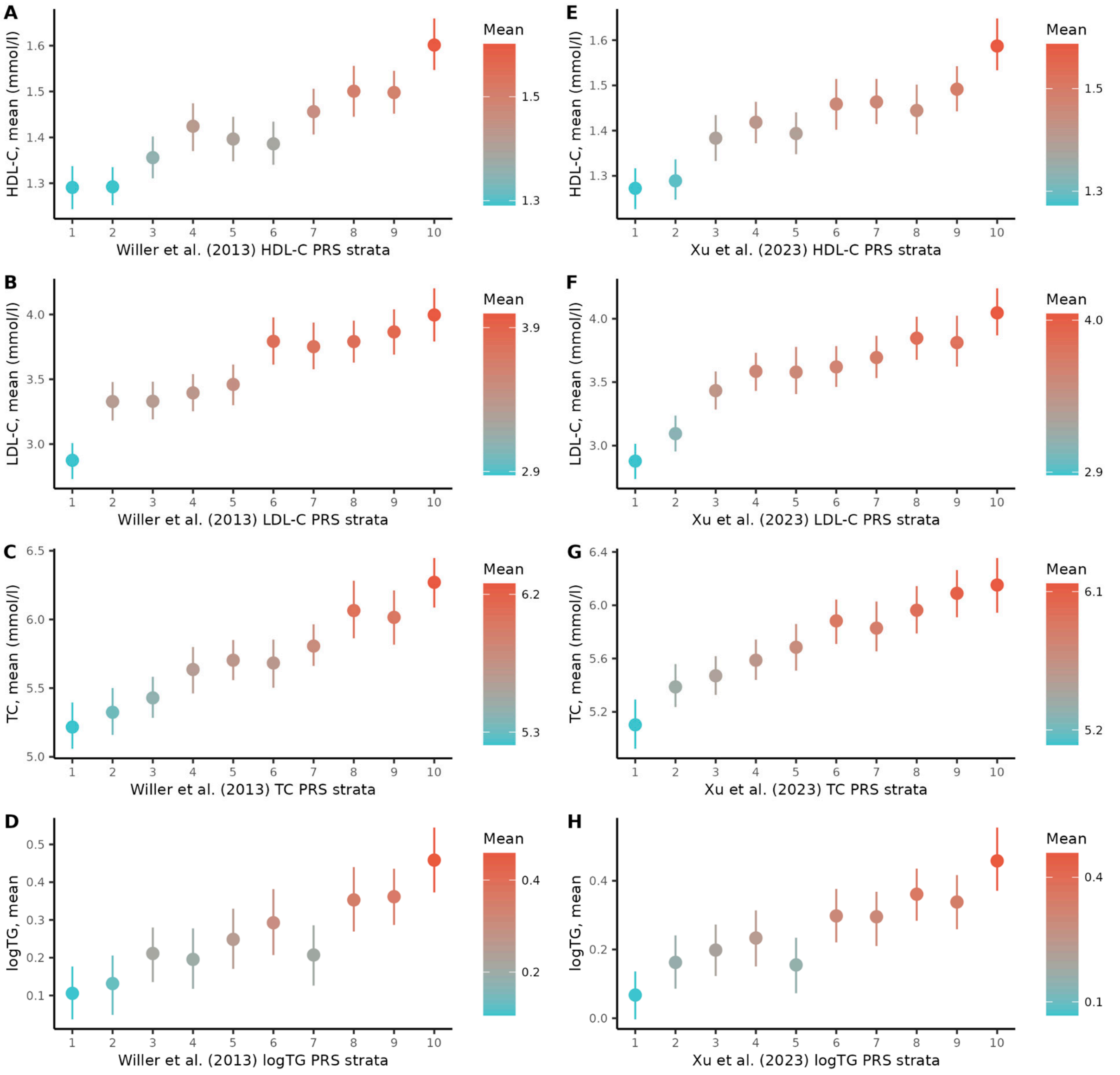

Figure S6

Distribution of mean lipid phenotype values by PRSice-2 PRS strata in ESSE-Vologda (train and validation dataset combined). Strata were calculated by sorting PRS z-scores from lowest to highest and splitting them into 10 equal parts. Scores A-D are from Willer et al. [2], scores E-H are from Xu et al. [1]. Scores A and E display the distribution of mean HDL-C levels (with 95% CI) in mmol/L across 10 PRS strata; scores B and F display the distribution of mean LDL-C levels (with 95% CI) in mmol/L across 10 PRS strata; scores C and G display the distribution of mean TC levels (with 95% CI) in mmol/L across 10 PRS strata; scores D and H display the distribution of mean logTG levels (with 95% CI) across 10 PRS strata. HDL-C — high-density

lipoprotein cholesterol; LDL-C — low-density lipoprotein cholesterol;, TC — total cholesterol; logTG — logarithmic triglycerides.

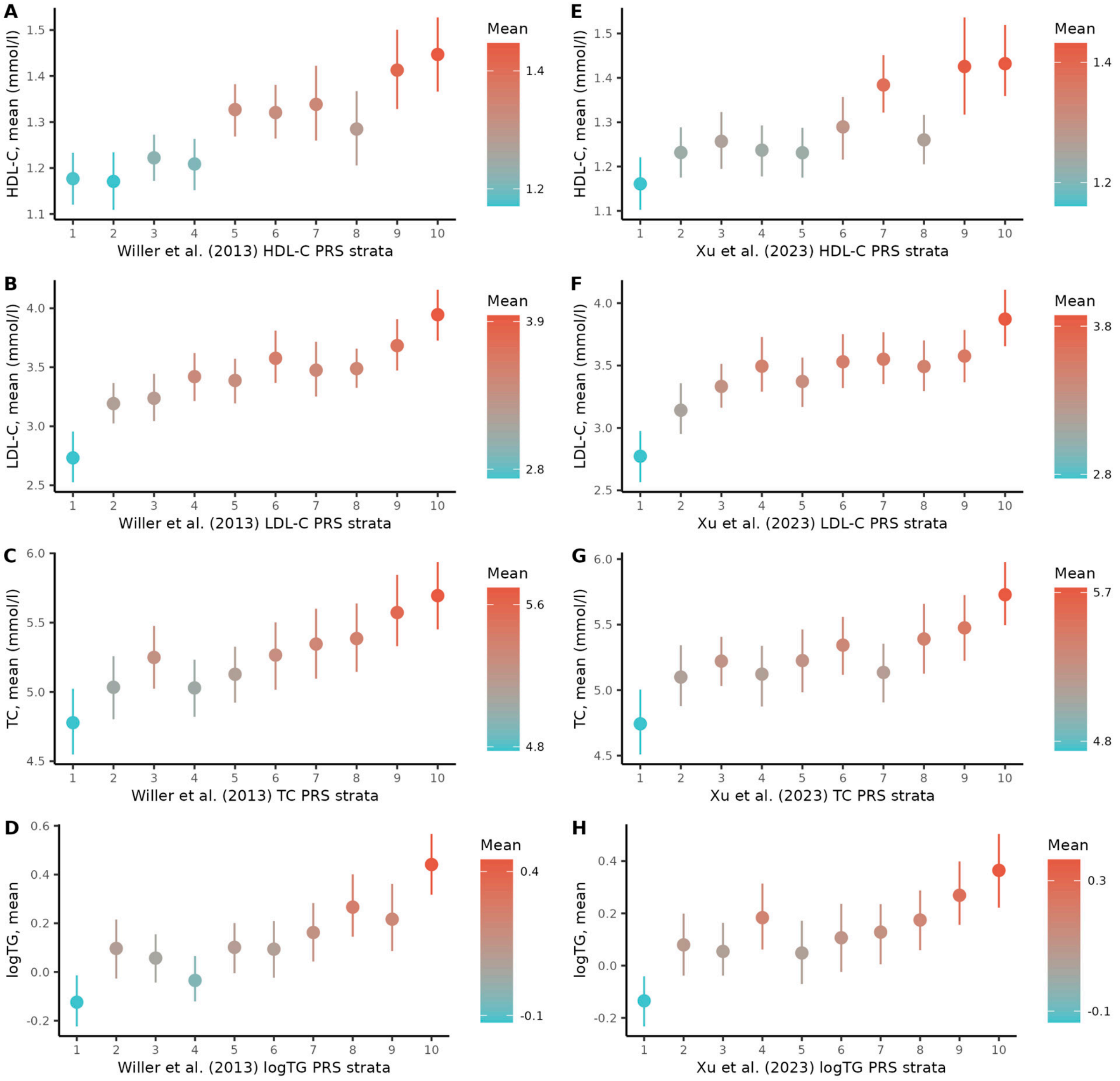

Figure S7

Comparison of Spearman's rho estimates across all developed scales. For PRSice-2, Spearman's rho estimates from both training and validation datasets are provided. Estimates are provided along with 95% CI. HDL-C—high-density lipoprotein cholesterol, LDL-C—low-density lipoprotein cholesterol, TC—total cholesterol, logTG—logarithmic triglycerides.

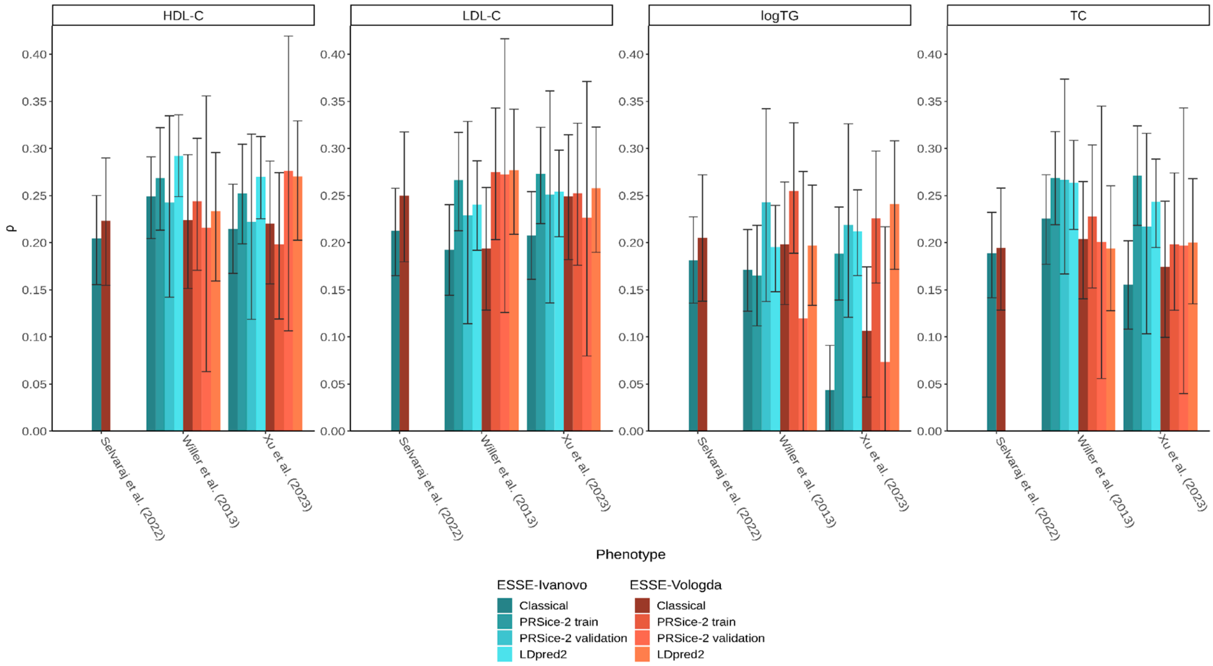

Figure S8

Distribution of mean lipid phenotype values by LDpred2 PRS strata in ESSE-Ivanovo. Strata were calculated by sorting PRS z-scores from lowest to highest and splitting them into 10 equal parts. Scores A-D are from Willer et al. [2], scores E-H are from Xu et al. [1]. Scores A, E display the distribution of mean HDL-C levels (with 95% CI) in mmol/L across 10 PRS strata; scores B and F display the distribution of mean LDL-C levels (with 95% CI) in mmol/L across 10 PRS strata; scores C and G display the distribution of mean TC levels (with 95% CI) in mmol/L across 10 PRS strata; scores D and H display the distribution of mean logTG levels (with 95% CI) across 10 PRS strata. HDL-C — high-density lipoprotein cholesterol; LDL-C — low-density lipoprotein cholesterol;, TC — total cholesterol; logTG — logarithmic triglycerides.

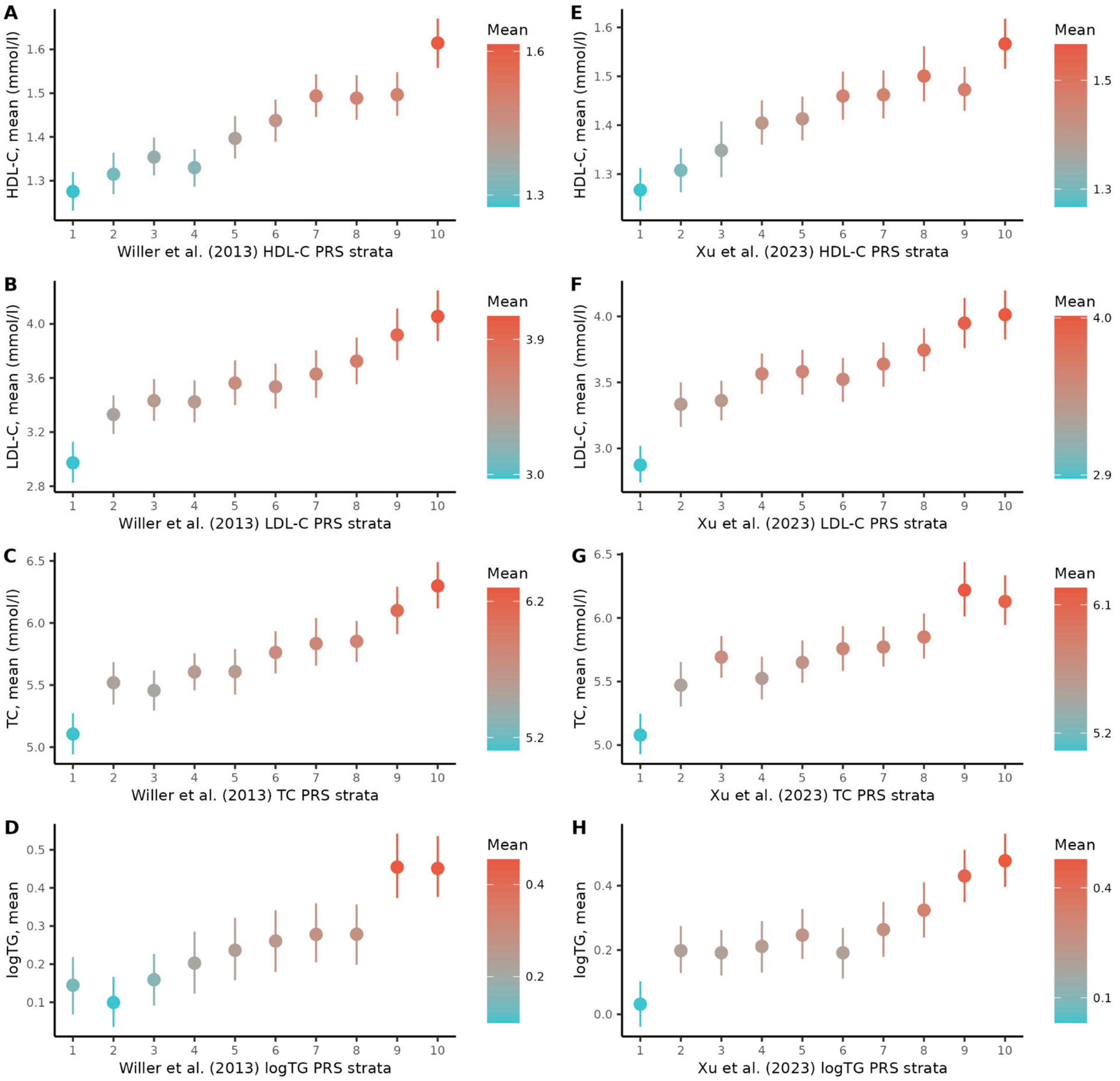

Figure S9

Distribution of mean lipid phenotype values by LDpred2 PRS strata in ESSE-Vologda. Strata were calculated by sorting PRS z-scores from lowest to highest and splitting them into 10 equal parts. Scores A-D are from Willer et al. [2], scores E-H are from Xu et al. [1]. Scores A and E display the distribution of mean HDL-C levels (with 95% CI) in mmol/L across 10 PRS strata; scores B, F display the distribution of mean LDL-C levels (with 95% CI) in mmol/L across 10 PRS strata; scores C and G display the distribution of mean TC levels (with 95% CI) in mmol/L across 10 PRS strata; scores D and H display the distribution of mean logTG levels (with 95% CI) across 10 PRS strata. HDL-C — high-density lipoprotein cholesterol, LDL-C —- low-density lipoprotein cholesterol, TC — total cholesterol, logTG — logarithmic triglycerides.

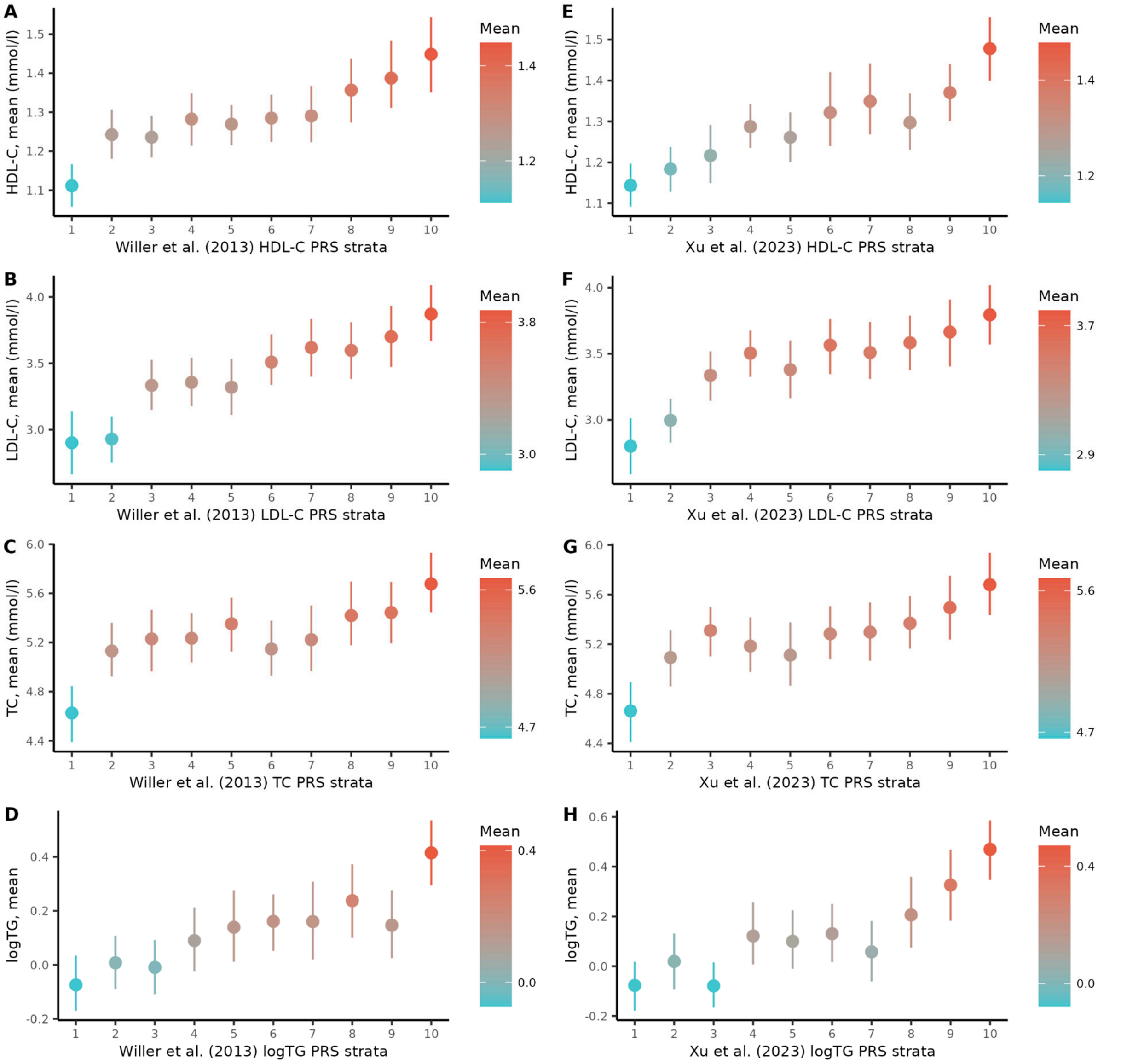

References

1. Xu, Y.; Ritchie, S.C.; Liang, Y.; Timmers, P.R.H.J.; Pietzner, M.; Lannelongue, L.; Lambert, S.A.; Tahir, U.A.; May-Wilson, S.; Foguet, C.; et al. An Atlas of Genetic Scores to Predict Multi-Omic Traits. *Nature* **2023**, *616*, 123–131, doi:10.1038/s41586-023-05844-9.

2. Willer, C.J.; Schmidt, E.M.; Sengupta, S.; Peloso, G.M.; Gustafsson, S.; Kanoni, S.; Ganna, A.; Chen, J.; Buchkovich, M.L.; Mora, S.; et al. Discovery and Refinement of Loci Associated with Lipid Levels. *Nat. Genet.* **2013**, *45*, 1274–1283, doi:10.1038/ng.2797.
3. Selvaraj, M.S.; Li, X.; Li, Z.; Pampana, A.; Zhang, D.Y.; Park, J.; Aslibekyan, S.; Bis, J.C.;

Brody, J.A.; Cade, B.E.; et al. Whole Genome Sequence Analysis of Blood Lipid Levels in >66,000 Individuals. *Nat. Commun.* **2022**, *13*, 5995, doi:10.1038/s41467-022-33510-7.
